# Effects of Daily Kombu (*Laminaria japonica*) Intake on Body Composition, Blood Pressure, and Fecal Microbiota in Healthy Adult Japanese: A Randomized, Double‐Blind Study

**DOI:** 10.1002/fsn3.70298

**Published:** 2025-05-19

**Authors:** Seiichiro Aoe, Hirofumi Ohtoshi, Fumiko Nakamura

**Affiliations:** ^1^ Department of Food Science, Faculty of Home Economics Otsuma Women's University Chiyoda‐ku Tokyo Japan; ^2^ Maruyanagi Oguraya Company Limited Kato‐shi Hyogo Japan; ^3^ CPCC Company Limited Chuo‐ku Tokyo Japan

**Keywords:** blood pressure, body fat, Japanese, kombu, microbiota

## Abstract

Visceral obesity is considered to have a significant role in the development of metabolic syndrome; therefore, a diet that suppresses visceral fat accumulation could prevent its onset. The effects of kombu consumption on reducing body fat and associated changes in gut microbiota were assessed in a randomized, double‐blind, placebo‐controlled intervention study in Japanese adults. Random assignment of study subjects between two groups was performed, and over 12 weeks the test group consumed cookies containing boiled kombu powder, while the placebo group consumed cookies containing cellulose. Anthropometric profiles for each participant, including visceral fat area (VFA), were recorded at weeks 0, 6, and 12. Body fat content and percentage were significantly decreased in the male kombu group compared to the placebo group. Systolic blood pressure was significantly lower in males and females in the kombu group compared with the placebo group. Serum adiponectin levels in males were significantly higher in the kombu group compared with the placebo group. The concentration of serum thyroid hormones did not increase after 420 μg/day of iodine consumption throughout kombu intake. Significant increases in the abundances of genus *Faecalibacterium*, *Bacteroides*, *Alistipes*, and *Agathobacter* were observed in the kombu group compared to the placebo group. Changes in body fat content and percentage were significantly negatively correlated with the relative abundance of *Agathobacter* in men. The consumption of kombu powder, which is high in alginate, may help prevent body fat accumulation in male subjects and improve microbiota in both males and females.

## Graphical abstract

The effects of kombu consumption on reducing body fat and associated changes in gut microbiota were assessed in a randomized, double‐blind, placebo‐controlled intervention study in Japanese adults. Body fat content and percentage were significantly decreased in the male kombu group compared to the placebo group. The consumption of kombu powder, which is high in alginate, may help prevent body fat accumulation in male subjects and improve microbiota in both males and females.

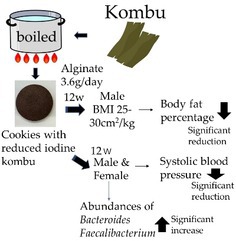

## 1 Introduction

Metabolic syndrome is a cardiovascular disease susceptibility state associated with insulin resistance, atherosclerosis‐inducing lipoprotein abnormalities, and high blood pressure (Eckel et al. 2005). The pathological conditions of metabolic syndrome include: (1) visceral fat accumulation, (2) insulin resistance, (3) atherosclerosis‐inducing lipoprotein abnormalities, (4) high blood pressure, and (5) other pathologies (Engin 2017). Of these, visceral obesity is thought to have a significant role in the development of metabolic syndrome (Matsuzawa et al. 2011). Therefore, a diet that suppresses the accumulation of visceral fat could prevent the onset of metabolic syndrome.

Kombu contains more dietary fiber than many other food ingredients, and in particular, sodium alginate, a water‐soluble dietary fiber, which is reported to have roles in health maintenance, such as lowering blood cholesterol and suppressing blood sugar levels (Jensen et al. 2013; Torsdottir et al. 1911). The blood cholesterol‐lowering effect of sodium alginate is thought to be due to the following mechanisms: (1) promotion of bile acid excretion and inhibition of reabsorption, (2) downregulation of cholesterol synthesis in the liver by acetate and propionate from microbiota fermentation, and (3) changes in cholesterol metabolism due to reduced insulin secretion. In addition, kombu intake reduces the rate of digestion and absorption of nutrients, so it is expected to be effective against obesity (Jensen et al. 2013; Kalita et al. 2022). Sodium alginate increases cholesterol excretion and glucose tolerance, and it was suggested that alginate, which converts to free alginic acid in the stomach, forms a highly viscous gel which inhibits cholesterol and glucose absorption from the small intestine (Kimura et al. 1996). We previously reported that 8 weeks of boiled kombu powder supplementation improved both weight loss and body fat percentage in overweight male subjects (3 g/day alginate intake) (Aoe et al. 2021). We also found that serum thyroid hormone levels were not significantly affected by additional supplementation of iodine in Japanese subjects with relatively high iodine intakes. However, there are no reports on whether a similar effect can be expected from kombu intake. In addition to sodium alginate, kombu contains laminarin and fucoidan as dietary fibers (Fauziee et al. 2021), so its effect on intestinal microbiota may differ from that of alginate extracts. This study investigated the effects of consuming cookies containing boiled kombu powder to replace staple foods over a long period of time (12 weeks) on body fat accumulation, as opposed to our previous report, which used tablet supplements. We also examined the effect on microbiota, of which there have been no reports to date on how kombu intake affects the microbiota in Japanese subjects.

## 2 Methods

### 2.1 Subjects

Our study abided by the regulations of the Institutional Review Board of Chiyoda Paramedical Care Clinic, and was run in concordance with the ethical guidelines for medical and health research involving human subjects (Ministry of Health, Labour and Welfare 2017) (IRB No. 15000088) and the Declaration of Helsinki. Before the study began, all subjects provided written informed consent. The primary objective of this study was to evaluate the effect of boiled kombu powder on reducing body fat and the associated changes in gut microbiota in subjects with high BMI.

The inclusion criteria were defined as:

1. Age 20–65 years.
2. BMI ≥ 23 kg/m^2^.
3. Regular eating habits, such as three meals a day.
4. A detailed description of the study was provided to all subjects, enabling a comprehensive understanding of the protocol and appropriate provision of written informed consent.

The exclusion criteria were defined as:

1. Those who regularly consumed foods (at least three times a week) for specified health uses or functional claims, or health foods (including supplements) that affect blood lipids or visceral fat, or foods high in dietary fiber and were unable to stop taking them from the time of obtaining consent.
2. Those taking medicines (lipid metabolism, laxatives, etc.) that may affect the study and who were unable to limit their intake during the study period.
3. Those who do not like kombu.
4. Those who drank a lot of alcohol (more than 60 g/day on average per week).
5. Those who refrained from or limited their intake of foods containing iodine.
6. Those with a history or current history of serious diseases of the heart, liver, kidneys, digestive system, etc.
7. Those who were pregnant, breastfeeding, or intended to become pregnant during the study period.
8. Those with allergies to medicines and foods.
9. Those participating in clinical trials of other drugs or health foods within 4 weeks of the end of the trial, or who planned to participate in another clinical trial after agreeing to participate in this trial.
10. Those who had donated blood components or 200 mL of whole blood from the month before the start of the trial.
11. Men who had donated 400 mL of whole blood 3 months before the start of the trial.
12. Women who had donated 400 mL of whole blood 4 months before the start of the trial.
13. Men whose blood volume collected from 12 months before the start of the trial exceeded 1200 mL when the planned total blood volume for this trial was added to their previously collected blood volume.
14. Women whose blood volume collected from 12 months before the start of the trial exceeded 800 mL when the planned total blood volume for this trial was added to their previously collected blood volume.
15. Those who the principal investigator or co‐investigator considered inappropriate to participate in this trial.

Forty‐four subjects—Japanese men or women—were randomly assigned to each trial group (kombu and placebo) stratified by VFA (primary outcome), BMI, age, sex, and serum LDL‐cholesterol level. A double‐blind, controlled, randomized trial was performed with the study protocol registered at the University Hospital Medical Information Network Clinical Trials Registry (UMIN000047577).

### 2.2 Preparation of Test Foods

Placebo and test cookie ingredients are shown in Table 1. Iodine was removed from the kombu (*Laminaria japonica*; Hokkaido Fisheries Corporative Associations, Hokkaido, Japan) by boiling for 60 min. The kombu was then dried and powdered. The alginate and iodine contents were determined by the Japan Food Research Laboratories (Tokyo, Japan): alginate was estimated using carbazole reagent in a colorimetric method (Udagawa et al. 2013), while iodine was analyzed using a gas chromatography—electron capture detector (GC‐ECD). The test food (kombu group) was a cookie containing 0.98 g of kombu powder per piece, and the control food (placebo group) was a cookie without kombu powder. Microcrystalline cellulose was added to the control food to ensure the same amount of dietary fiber. Each cookie contained 0.6 g of alginate and 0.07 mg of iodine. The placebo and test cookies were identical in appearance (Figure S1).

**TABLE 1:** Test cookie components (g/piece).

	Placebo	Kombu
Boiled kombu powder	0	0.98
Maltitol	3.25	3.25
Microcrystalline cellulose	1.69	0
Cake flour	3.00	3.70
Unsalted butter	3.25	3.25
Whole egg	1.00	1.00
Cocoa powder	0.78	0.78
Baking powder	0.04	0.04

*Note:* One test cookie contained 0.07 mg iodine and 0.6 g alginate.

### 2.3 Study Design and Intervention

The trial participants and researchers were all unaware of which group they had been assigned to. Subjects were asked to consume six cookies per day for 12 weeks, replacing the staple food for either breakfast, lunch, or dinner. The replacement cookies were the equivalent of approximately one cup of rice (200 g) or two slices of bread (6 slice type). If subjects were unable to finish the test cookies in one meal, they were allowed to split them into two meals. Alginate dose was based on a previous report, which used 3.28 g/day of alginate supplements in healthy Japanese adults (Aoe et al. 2021). Throughout the trial, subjects recorded daily intake amounts and the time of intake for each test food. At week 0 (before intervention) and week 12 (final week of the trial), subjects also reported on their daily activity and provided a dietary record detailing everything they had consumed over three weekdays and 1 day off. The Standard Tables of Food Composition in Japan 2020, 8th Revised Version (Ministry of Education, Culture, Sports, Science and Technology 2020), in combination with Excel Eiyokun Version 8 software (Kenpakusha, Tokyo, Japan), were used to analyze food records. Dashi provides the stock ingredient for Japanese soup: it contains kombu extract and provides the majority of iodine in the Japanese diet. However, there is significant variation in the amount of iodine present, depending on which soup stock or miso is used. If soup was listed as a consumed food, we assumed it was made using a Japanese granule soup stock and light‐colored spicy miso and calculated iodine intake accordingly. Subject data was excluded if they did not consume all 6 cookies every day or their intake diary was not recorded according to the study protocol for more than 85% of the trial. Body weight, height, body fat percentage, visceral fat area, and blood samples, taken from a forearm vein, were collected at baseline and in the final week of the trial. Fecal samples were collected using a stool collection container (Metabolokeeper, TechnoSuruga Laboratory, Japan), containing guanidine thiocyanate and a detergent containing ethylenediaminetetraacetic acid disodium salt dihydrate (EDTA) 2Na dihydrate, tris (hydroxymethyl)aminomethane, and sodium diphosphate before transfer to the laboratory at room temperature.

### 2.4 Clinical Analyses

The InBody570 body composition analyzer (InBody Japan Inc., Tokyo, Japan) was used for body weight and body fat percentage analysis. A Dual Scan (HDS‐2000; Omron Healthcare Co. Ltd., Kyoto, Japan) measured visceral fat areas. Total fat and visceral fat areas were determined for each axial tomographic slice. Body weight and height were measured and used to calculate BMI. All urine and blood samples, collected after an overnight fast, were analyzed by LSI Medience Corporation (Tokyo, Japan). Serum thyroid hormone levels (thyroid‐stimulating hormone [TSH], thyroxine [FT4], triiodothyronine [FT3]), which are indicative of thyroid function, were determined using Chemilumi‐ACS‐TSH, Chemilumi‐ACS‐FT3, and Chemilumi‐ACS‐FT4 kits, respectively (Roche Diagnostics, Indianapolis, IN, USA). Enzyme‐linked immunosorbent assay (ELISA) kits (Quantikine ELISA kit; R&D Systems, Minneapolis, MN) quantified serum leptin and adiponectin concentrations.

### 2.5 DNA Extraction and Composition Analysis of Intestinal Microbiota Using High‐Throughput Sequencing

DNA extractions were performed as described previously (Takahashi et al. 2014), using an automated DNA isolation system (GENE PREP STAR PI‐480 Kurabo, Japan). Pro341F/Pro805R primers amplified the V3–V4 regions of bacterial and archaeal 16S rDNA, using the dual‐index method (Takahashi et al. 2014; Hisada et al. 2015). The MiSeq Reagent Kit, version 3 (600 Cycle) chemistry, was employed for paired‐end sequencing of barcoded amplicons (2 × 301‐bp) using the MiSeq system. Primer sequences were trimmed from the paired‐end sequencing reads by cutadapt ver 1.18, with default settings (Martin 2011). The fastq‐join program merged the paired‐end sequencing reads (default settings) (Aronesty 2013). Merged reads with a quality value score of ≥ 20 for greater than 99% of the sequence were extracted using the FASTX‐Toolkit (Gordon and Hannon 2020). Chimeric sequences were removed after detection by usearch61 (Edgar et al. 2011). The Ribosomal Database Project (RDP) Classifier ver 2.13 (Wang et al. 2007) and TechnoSuruga Lab Microbial Identification database (DB‐BA) ver 16.0 (TechnoSuruga Laboratory, Japan) were used for taxonomy classification (Kasai et al. 2015). Identification of 16S/28S/ITS rRNA genes was enabled by Metagenome@KIN ver 2.2.1 analysis software (World Fusion, Japan), at confidence ≥ 0.8 and homology of ≥ 97% for the RDP Classifier and DB‐BA databases, respectively.

QIIME2 ver 2020.6 was used to process joined amplicon sequence reads (Bolyen et al. 2019). Quality filtering and chimeric sequence deletion were performed before DADA2 (Divisive Amplicon Denoising Algorithm 2) denoise‐single ver 1.10.0 (default settings) was used to create representative sequences (Callahan et al. 2016). The SILVA database ver 138 (Quast et al. 2013), trained using a Naive Bayes classifier, created taxonomy information for representative sequences. The samples were rarefied to a minimum of 25,293 sequences per sample before alpha diversity indices (chao1, shannon, and simpson) and beta diversity metrics (weighted UniFrac, unweighted UniFrac, and Bray–Curtis distances) were determined. A 2D principal coordinate analysis (2D‐PCoA) was also performed using qiime2R ver 0.99.13 (Jordan 2018) and tidyverse ver 1.2.1 (Wickham 2017) libraries in R (R Core Team 2017). The statistical significance of chao1, shannon, and simpson indices among groups was determined using the Kruskal–Wallis test. The ANOSIM test was used to measure the statistical significance of the similarity of bacterial communities among groups.

### 2.6 Fecal Organic Acids

Fecal viruses were inactivated by heating fecal: distilled water suspensions to 85°C for 15 min before centrifugation, as reported previously (Higashimura et al. 2016). Fecal concentrations of: formic acid, acetic acid, propionic acid, isobutyric acid, *n*‐butyric acid, valeric acid, isovaleric acid, lactic acid, and succinic acid were determined using high‐performance liquid chromatography (HPLC) organic acid analysis, with a Prominence CDD‐10A conductivity detector (Shimadzu, Kyoto, Japan). The columns consisted of Shim‐pack SCR‐102 (H) (300 mm × 8 mm inner diameter [ID]) for separation, and a Shim‐pack SCR‐102 (H) (50 mm × 6 mm ID) as a guard column (Zhang et al. 2008). Organic acids were measured using an HPLC calibration curve using standard solutions.

### 2.7 Statistical Analyses

Based on our previous report, sample sizes were determined to require > 18 subjects per group (type I error [*α*] = 0.05, 1 − *β* = 0.80) (Aoe et al. 2021). A quantile–quantile plot verified data normality. Bartlett's test was used to estimate homogeneity of variances. A JMP statistical software package (Version 16.0; SAS Institute Inc., Cary, NC, USA) was used to evaluate the data. ANCOVA analyzed the differences between the changes from baseline at each time period for the placebo and kombu groups: the baseline was the covariate if the two groups had equal slopes in the regression of each period data on baseline data. Comparisons between baseline and each time point in each group were analyzed using Dunnett's multiple comparisons test. Comparisons between two groups at each time point were performed using the Bonferroni multiple comparisons test (significance adjusted to *p* < 0.166). Measurements were also stratified by gender if gender differences were observed. Correlations between increased microbiota abundance after test cookie consumption and body fat percentage and fat mass were calculated using Pearson's correlation coefficient and significance level. A two‐sided *p*‐value < 0.05 was considered significant throughout data analyses.

## 3 Results

### 3.1 Subject Characteristics

**FIGURE 1:**
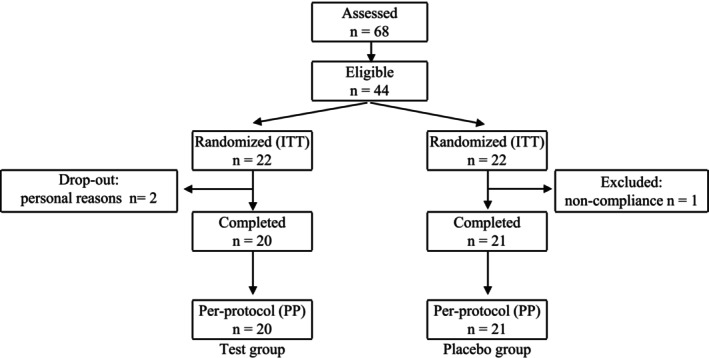
Flow diagram of subjects throughout the 12‐week intervention study (ITT, intention to treat).

Forty‐four subjects were eligible for trial participation. Three subjects dropped out during the study: two subjects in the placebo group and one subject in the kombu group withdrew for reasons unrelated to the study (Figure 1). The remaining participants completed the trial. The baseline characteristics of the subjects are shown in Table 2. The two groups were similar in age, height, body weight, and body mass index. Serum chemistry values are shown in Table S1. After 12 weeks of test food consumption, LDL‐ and HDL‐cholesterol concentrations were significantly reduced compared to baseline levels in both groups, but no between‐group differences were observed. Serum uric acid concentrations increased significantly compared to baseline levels in the placebo group, but there were no significant concentration differences in the kombu group. Several other measured parameters showed significant changes from the baseline; however, during the intervention period, no adverse or abnormal changes in measured blood parameters were observed between the two groups. Thirty‐eight subjects had a 100% intake rate of the test food, while 3 subjects recorded their intake at greater than 85%. During the study, one subject experienced loose stools due to the test food, and the subject declined to continue the study. The remaining subjects were included in data analysis. During the study period, no adverse events (e.g., gastrointestinal problems) were found to be related to the consumption of kombu cookies, except for one subject who was excluded. Statistical analyses of data from subjects in the placebo (*n* = 20) and kombu (*n* = 21) groups were performed according to the per‐protocol set.

**TABLE 2:** Baseline characteristics of subjects.

Group	Gender	*n*	Age (years)	Height (cm)	Body weight (kg)	BMI (kg/m^2^)	Systolic blood pressure (mmHg)	Diastolic blood pressure (mmHg)
Placebo	Male	12	49.0 ± 1.8	171.7 ± 1.5	77.8 ± 1.6	26.4 ± 0.4	126.7 ± 4.9	87.3 ± 3.0
	Female	8	51.8 ± 5.9	160.1 ± 1.7	66.1 ± 1.4	25.8 ± 0.4	127.5 ± 4.4	80.3 ± 3.0
Kombu	Male	14	49.9 ± 2.3	168.9 ± 1.5	75.4 ± 1.9	26.4 ± 0.6	127.5 ± 3.9	83.0 ± 3.2
	Female	7	48.7 ± 10.3	158.1 ± 2.0	65.4 ± 2.5	26.1 ± 0.5	129.6 ± 4.2	80.0 ± 2.9

*Note:* Mean ± standard error (SE). There were no statistically significant differences between the two groups.

### 3.2 Energy and Nutrient Intake

Throughout the intervention period, the intakes of energy and nutrients for each subject were recorded and are presented in Table 3. No significant differences in calorie or nutrient (protein, fat, carbohydrate, total dietary fiber, and iodine) intakes were observed between the placebo and kombu groups at baseline or during intervention.

**TABLE 3:** Daily energy and nutrient intake during the trial.

Week		0 W	12 W^a^
Energy (kcal)	Placebo	1757 ± 73	1470 ± 75
	Kombu	1897 ± 93	1506 ± 100
Protein (g)	Placebo	67.4 ± 3.2	60.7 ± 3.9
	Kombu	74.7 ± 3.8	62.7 ± 3.0
Fat (g)	Placebo	55.4 ± 3.4	52.2 ± 4.2
	Kombu	62.3 ± 3.9	54.8 ± 4.5
Carbohydrate (g)	Placebo	258.9 ± 11.2	198.8 ± 10.7
	Kombu	275.0 ± 14.8	200.0 ± 14.1
Total dietary fiber (g)	Placebo	18.7 ± 0.9	15.4 ± 0.9
	Kombu	18.4 ± 0.7	14.1 ± 0.6
Iodine (μg)	Placebo	2312 ± 803	2368 ± 462
	Kombu	2906 ± 743	2222 ± 590

*Note:* Mean ± standard error (SE). Average daily record for 3 days. There were no statistically significant differences between the two groups.

^a^ The dietary survey results do not include energy and nutrients derived from the test cookies, which were 300 kcal/day.

### 3.3 Anthropometric, Visceral and Subcutaneous Fat Area Analysis

**FIGURE 2:**
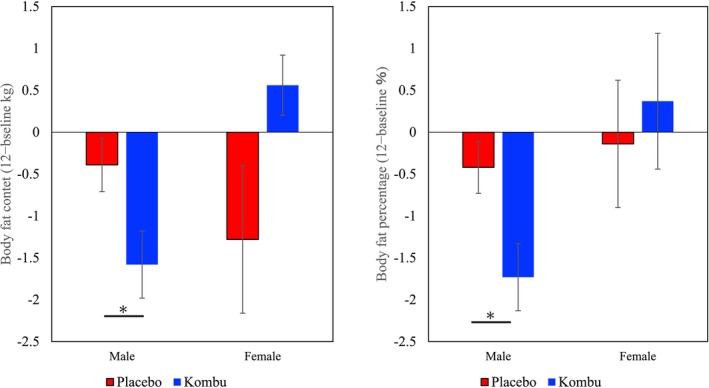
Effects of boiled kombu powder on body fat content and body fat percentage (bars represent means and standard error [SE]). *Significantly different from the placebo group (*p* < 0.05).

Table 4 and Figure 2 show gender‐stratified changes in anthropometric, visceral, and subcutaneous fat area measurements after consumption of boiled kombu powder for 12 weeks. After 12 weeks, the decreases in body fat content and percentage were significantly greater in males in the kombu group compared with the placebo group. Compared with baseline (0 weeks), body fat content and body fat percentage were significantly reduced in the male kombu group only at both 6 and 12 weeks, whereas body fat percentage was significantly higher in the female kombu group compared with the female placebo group. Compared with baseline (0 weeks), body weight, visceral fat area, and waist circumference were significantly increased in the female kombu group at week 6, but these changes were temporary. Compared with week 0, waist circumference was significantly reduced in the female placebo group at week 12, and there were no significant differences between the placebo and kombu group in visceral or subcutaneous fat area measurements.

**TABLE 4:** Effect of boiled kombu powder on anthropometric measurements and visceral and subcutaneous fat area.

	Gender	Placebo			Kombu		
		Baseline	6 W	12 W	Baseline	6 W	12 W
Body weight (kg)	Male	77.8 ± 1.6	77.6 ± 1.4	77.6 ± 1.6	75.4 ± 1.9	74.4 ± 2.0	74.4 ± 2.0
	Female	66.1 ± 1.4	65.4 ± 1.8	64.8 ± 2.1	65.4 ± 2.5	66.1 ± 2.4	66.2 ± 2.4*
BMI (kg/m^2^)	Male	26.4 ± 0.4	26.3 ± 0.4	26.3 ± 0.4	26.4 ± 0.4	26.3 ± 0.6	26.1 ± 0.6
	Female	25.8 ± 0.4	25.5 ± 0.6	25.3 ± 0.7	26.1 ± 0.5	26.4 ± 0.4	26.4 ± 0.4
Body fat content (kg)	Male	21.9 ± 1.3	21.5 ± 1.3	21.5 ± 1.3	21.8 ± 1.2	20.7 ± 1.2	20.3 ± 1.1*^,^**
	Female	24.0 ± 0.8	23.1 ± 1.6	22.7 ± 1.4	25.7 ± 1.3	26.1 ± 1.3	26.3 ± 1.2
Body fat percentage (%)	Male	28.0 ± 1.4	27.6 ± 1.4	27.6 ± 1.5	28.8 ± 0.9	27.6 ± 0.9*	27.1 ± 0.8*^,^**
	Female	36.3 ± 1.1	35.3 ± 1.2	34.9 ± 1.4	39.3 ± 3.2	39.6 ± 1.1	39.7 ± 1.1
Visceral fat area (cm^2^)	Male	92.0 ± 5.5	91.4 ± 5.2	90.2 ± 5.4	88.5 ± 7.6	87.7 ± 8.2	83.1 ± 8.8
	Female	59.5 ± 2.9	65.4 ± 4.6	61.2 ± 6.5	59.4 ± 2.9	65.5 ± 4.7	60.0 ± 3.0
Subcutaneous fat area (cm^2^)	Male	209.0 ± 12.7	213.1 ± 11.6	204.6 ± 11.8	214.4 ± 9.8	216.8 ± 13.7	206.5 ± 13.4
	Female	264.6 ± 19.9	253.6 ± 19.9	243.8 ± 23.9	278.9 ± 18.6	29.38 ± 22.4	293.0 ± 15.3
Waist circumference (cm)	Male	94.0 ± 1.4	94.2 ± 1.1	93.7 ± 1.4	94.9 ± 1.7	94.3 ± 1.7	93.7 ± 1.8
	Female	94.9 ± 1.8	93.3 ± 2.3	92.2 ± 2.4*	93.2 ± 1.2	95.6 ± 1.7	94.6 ± 1.8

*Note:* Mean ± standard error (SE). No significant differences between the placebo and kombu groups were observed at baseline, 6 W, and 12 W (Bonferroni's multiple comparison).

*p < 0.05 (ANCOVA baseline as a covariate); **p < 0.05 (vs. baseline, Dunnett's multiple comparison).

**FIGURE 3:**
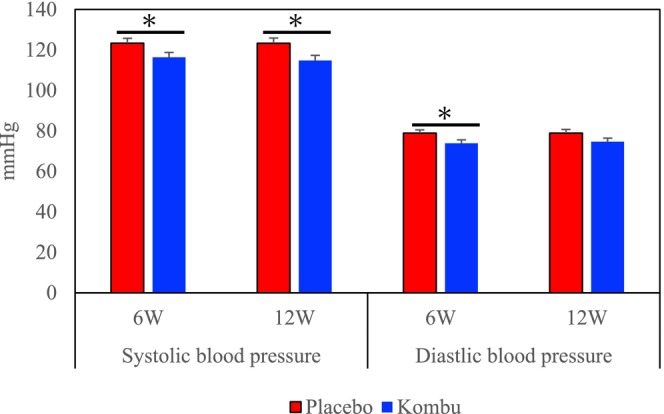
Effects of boiled kombu powder on systolic and diastolic blood pressure (bars represent means and standard error [SE]). *Significantly different from the placebo group (*p* < 0.05).

Systolic and diastolic blood pressures are shown in Figure 3. ANCOVA showed that systolic blood pressure was significantly reduced in the kombu group compared to the placebo group at weeks 6 and 12 in both genders. ANCOVA also showed that diastolic blood pressure was significantly reduced in the kombu group compared to the placebo group at week 6 in both genders.

### 3.4 Serum Adiponectin and Leptin Concentrations

**FIGURE 4:**
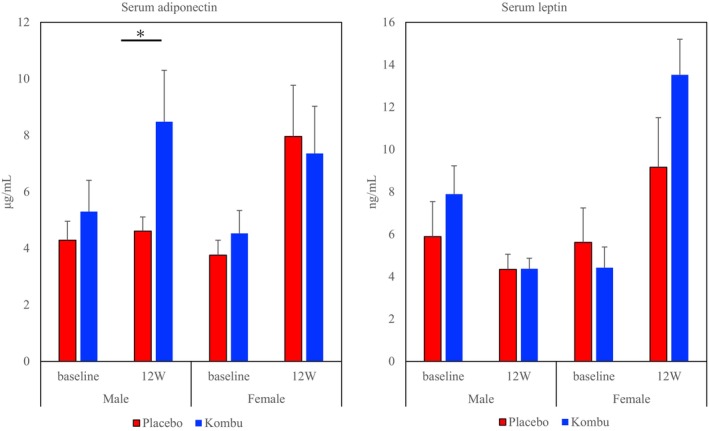
Effects of boiled kombu powder on serum adiponectin and leptin concentrations (bars represent means and standard error [SE]). *Significantly different from the placebo group (*p* < 0.05).

The concentrations of serum leptin and adiponectin are shown in Figure 4. The male kombu group showed significantly higher serum adiponectin concentrations compared to the placebo group by ANCOVA, but no significant changes were observed in the female kombu group. Serum leptin concentrations did not significantly differ between the placebo and kombu groups.

### 3.5 Serum Thyroid Hormone Concentrations

No significant differences in serum thyroid hormone concentrations (TSH, FT3, and TF4) were observed between the test groups or during the trial (Table 5). The levels of TSH, FT3, and TF4 were not significantly altered after 420 μg/day of iodine supplementation obtained by kombu intake.

**TABLE 5:** Effect of boiled kombu powder on TSH and thyroid hormone concentrations.

	Placebo			Kombu		
	Baseline	12 W	12 W‐baseline	Baseline	12 W	12 W‐baseline
TSH (μIU/mL)	1.65 ± 0.18	1.89 ± 0.23	0.25 ± 0.19	1.85 ± 0.22	2.01 ± 0.22	0.05 ± 0.29
FT3 (pg/mL)	3.13 ± 0.08	3.10 ± 0.09	−0.04 ± 0.07	2.97 ± 0.08	2.94 ± 0.08	−0.17 ± 0.16
FT4 (ng/dL)	1.21 ± 0.03	1.22 ± 0.03	−0.01 ± 0.03	1.26 ± 0.04	1.25 ± 0.04	−0.09 ± 0.07

*Note:* Mean ± standard error (SE). There were no statistically significant differences between the two groups.

Abbreviations: FT3, triiodothyronine; FT4; thyroxine; TSH, thyroid‐stimulating hormone.

### 3.6 Fecal Microbiota and Fecal SCFAs Concentrations

**FIGURE 5:**
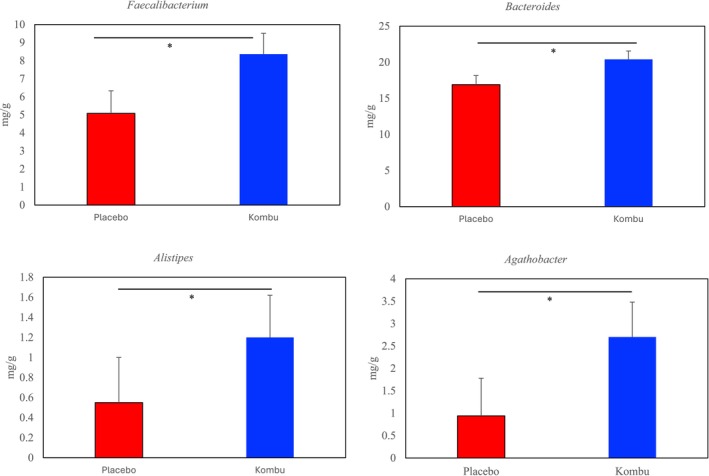
Effects of boiled kombu powder on fecal microbiota at genus level (bars represent means and standard error [SE]). *Significantly different from the placebo group (*p* < 0.05).

Figure S1 shows the changes in the intestinal flora at the phylum level before and after test food consumption (0 W and 12 W). After 12 weeks, Bacteroidota abundance decreased, while the abundances of Firmicutes and Actinobacteriota increased; however, there were no differences at the phylum level between groups at week 12. Using the 0 W abundance of genera at 1% or higher in the intestinal flora of feces as a covariate, *Faecalibacterium*, *Bacteroides*, *Alistipes*, and *Agathobacter* were significantly higher in the kombu group (Figure 5). Pearson's correlation coefficients between increased microbiota abundance after test cookie consumption and body fat percentage and fat mass are shown in Table 6. A significant negative correlation was observed between the abundance of *Agathobacter* and body fat percentage and fat mass in male subjects.

**TABLE 6:** Pearson's correlation coefficient between body fat and microbiota.

Male	Gender	Body fat content (12 W‐baseline)	Body fat percentage (12 W‐baseline)
*Faecalibacterium*	Male	0.0304	−0.1368
	Female	−0.1964	−0.1613
*Bacteroides*	Male	−0.0851	0.0716
	Female	0.1727	0.1112
*Alistipes*	Male	−0.1231	−0.0904
	Female	0.1914	0.1634
*Agathobacter*	Male	−0.398*	−0.4553*
	Female	−0.0915	−0.1371

* *p* < 0.05.

After 12 weeks, the change in Simpson's alpha diversity index was significantly greater in the kombu group compared with the placebo group (Table 7). No significant differences were detected between the groups in other alpha diversity indices or beta diversity (data not shown).

**TABLE 7:** Effect of boiled kombu powder on α‐diversity index of fecal microbiota.

	Placebo			Kombu		
	Baseline	12 W	12 W‐baseline	Baseline	12 W	12 W‐baseline
Shannon	5.32 ± 0.13	5.57 ± 0.12	0.25 ± 0.07	4.98 ± 0.17	5.41 ± 0.15	0.43 ± 0.09
Shao1	179.2 ± 16.5	146.8 ± 8.9	−33.3 ± 10.4	157.1 ± 11.7	135.1 ± 8.4	−22.0 ± 4.9
Simpson	0.94 ± 0.01	0.96 ± 0.01	0.02 ± 0.00	0.91 ± 0.02	0.95 ± 0.01	0.04 ± 0.01*
Observed OTUs	177.7 ± 16.2	145.8 ± 8.7	−31.9 ± 10.3	156.3 ± 11.6	134.3 ± 8.3	−22.0 ± 4.9
Faith PD	15.5 ± 1.2	14.2 ± 0.7	−1.30 ± 0.87	14.3 ± 0.9	13.6 ± 0.8	−0.68 ± 0.79

*Note:* Mean ± standard error (SE).

Abbreviations: Faith PD, Faith's phylogenetic diversity; Observed OTUs, observed operational taxonomic units.

* Significantly different from the placebo group (*p* < 0.05).

Fecal SCFAs concentrations are shown in Figure S2. The major SCFAs were acetate, propionate, and *n*‐butyrate; however, no significant differences were detected between the test and placebo groups.

## 4 Discussion

A parallel, double‐blind, placebo‐controlled trial was performed to investigate the effect of supplemental intake of boiled kombu powder on body composition in Japanese adults. Healthy subjects were randomly allocated into two groups, which received either a placebo cookie or a boiled kombu cookie (alginate 3.6 g/day) group. A significant reduction in body fat content and percentage was observed in male subjects within the kombu cookie group compared to the placebo group after 12 weeks consumption. Moreover, by the final week of the trial (week 12) there was significantly higher abundance of beneficial microbiota, such as the genus *Faecalibacterium*, *Bacteroides*, *Alistipes*, and *Agathobacter* in the kombu group compared to the placebo group. This is the first report showing that kombu intake improves intestinal microbiota in Japanese people.

We previously reported safe and significant reductions in body fat percentage in overweight male subjects after 8 weeks consumption of iodine‐reduced kombu powder (Aoe et al. 2021). It was concluded that, in Japanese subjects who consume relatively high concentrations of iodine, dietary supplementation with kombu high in alginate may help prevent body fat accumulation without affecting thyroid function. The body fat‐reducing effect was only seen in males, so we aimed to verify whether the same effect would be seen in females if kombu was ingested for more than 8 weeks. In addition, we investigated whether the effects of kombu differ when ingested as a food replacement or when taken as a tablet after meals, as in the previous study.

The incidence of both cardiovascular disease and obesity differs between men and women (Link and Reue 2017). Generally, the accumulation of visceral adipose tissue tends to be greater in men compared with women. In contrast, increases in subcutaneous adipose tissue tend to be observed in women; this increases fat mass in proportion to body weight. The incidence of metabolic syndrome is much lower in Japanese women compared to Japanese men (14.3% in women, 25% in men) (Kokubo et al. 2008). In this study, a decrease in body fat content and percentage after 12 weeks consumption of test cookies containing boiled kombu powder was only seen in male subjects. This result is consistent with the results of a previous study (Aoe et al. 2021): kombu is effective in reducing body fat in adults, especially in men with excess body fat, when ingested as a tablet or a meal. The mechanism of gender differences needs to be verified in future experiments.

The kombu group had significantly higher levels of serum adiponectin compared to the placebo group at 12 weeks in male subjects. It was previously reported that serum adiponectin concentration decreases with obesity, and this decrease is involved in the exacerbation of metabolic syndrome. The high serum adiponectin levels in the male subjects in this study are thought to be related to a decrease in body fat mass.

Increases in the abundances of *Faecalibacterium* and *Bacteroides* genera in the kombu group are thought to be an improvement towards favorable enterotypes. In a previous study, human gut conditions were mimicked using fecal samples from Japanese volunteers, and the results suggested alginate products, produced after assimilation by *Bacteroides* species, may encourage the growth of *Faecalibacterium prausnitzii* (Murakami et al. 2021). The results of our human intervention study confirmed these in vitro findings. Takagi et al. classified the enterotypes of the microbiota of Japanese people into five types (Takagi et al. 2022). Among them, types with a high proportion of *Bacteroides* and *Faecalibacterium* have been reported to be healthy enterotypes with fewer diseases. Levels of butyrate‐producing bacteria, *Faecalibacterium* and *Agathobacter*, increased with kombu consumption. In the human large intestine, the main butyrate‐producing bacteria are *Faecalibacterium*. Butyrate is reported to promote cell differentiation, cell cycle arrest, and apoptosis of transformed colonocytes and may have a preventive effect against colon cancer (Wong et al. 2006). In addition, butyrate inhibits histone deacetylase and reduces primary bile acid conversion to secondary bile acids (Wong et al. 2006; Zeng et al. 2019). Therefore, the increase in butyrate‐producing bacteria caused by kombu consumption may protect against colonic diseases. However, no difference was observed in fecal butyrate concentrations between the groups. It is thought that the difference could not be detected in feces because the timing of stool collection could not be controlled: butyrate is rapidly absorbed and utilized in the large intestine. To resolve this, we are currently investigating the SCFA production ability of boiled kombu using an in vitro fermenter. In addition, the proportion of *Alistipes* bacteria was significantly higher with kombu intake. The results of a recent integrated omics analysis, which comprehensively examined the intestinal bacteria and fecal metabolites of 306 Japanese people, reported that *Alistipes* bacteria have an effect on improving insulin resistance (Takeuchi et al. 2023). Therefore, it is expected that kombu intake will improve insulin resistance. An inverse correlation was observed between the abundance of *Agathobacter* and the percentage and content of body fat. *Agathobacter rectale*, a bacterium renamed *Eubacterium rectale* , is a butyric acid‐producing bacterium. Although there are no reports of its effect on inhibiting body fat accumulation, it may be a related bacterium that reduces body fat. However, there was very little difference in *Agathobacter* abundance between the placebo and kombu groups, and the decrease in body fat cannot be explained by changes in a specific bacterial genus alone. Kombu contains sodium alginate, a highly viscous water‐soluble dietary fiber, and we previously suggested that the main mechanism in decreasing body fat percentage after kombu ingestion is the inhibition of carbohydrate and lipid absorption (Aoe et al. 2021).

Simpson's index increased with kombu consumption. It was previously reported that supplementation with a single fermentable dietary fiber significantly increased certain gut bacteria, and therefore reduced diversity indices (Cheng et al. 2017). Since kombu contains multiple fermentable dietary fibers, it is suggested that it improves gut microbiota without reducing alpha diversity. The abundance of several bacterial genera increased during 12 weeks of consumption of test cookies in place of staple foods. *Lachnospiraceae*, *Agathobacter*, *Anaerostipes*, *Blautia*, *Lachnoclostridium*, *Roseburia*, and *Eubacterium*, which belong to the phylum Firmicutes, all increased by several percent to 10%. While the abundance of the phylum Firmicutes increased, the abundance of the phylum Bacteroidota decreased. The small sample size is one of the major limitations of this study. We plan to complete a larger follow‐up study to investigate the effect of boiled kombu on visceral fat loss. These results showed that kombu intake had only a limited effect on preventing visceral obesity, as no significant reduction in visceral fat area was observed. Therefore, further research is needed to demonstrate the preventive effect of kombu intake on lifestyle‐related diseases.

## 5 Conclusions

The consumption of boiled kombu (alginate 3.6 g/day) for 12 weeks was found to promote body fat loss and increase serum adiponectin levels in moderately obese male adult subjects. In addition, this is the first report that kombu consumption improves the microbiota in Japanese men and women.

## Author Contributions

**Seiichiro Aoe:** conceptualization (lead), methodology (lead), writing – original draft (lead), writing – review and editing (lead). **Hirofumi Ohtoshi:** conceptualization (supporting), resources (supporting), writing – review and editing (supporting). **Fumiko Nakamura:** conceptualization (supporting), methodology (equal), writing – review and editing (supporting).

## Ethics Statement

The study protocol was approved by Chiyoda Paramedical Care Clinic according to the ethical guidelines for medical and health research involving human subjects (IRB No. 15000088).

## Consent

Written consent from all subjects was taken and ethical approval was obtained from Chiyoda Paramedical Care Clinic.

## Conflicts of Interest

The authors declare no conflicts of interest.

## Supporting information


**Figure S1.** Appearance of the test cookies.
**Figure S2.** The average relative abundances of detected bacteria phylum and genera in stool samples obtained at baseline and week 12.
**Figure S3.** Fecal SCFA concentrations of subjects at baseline and week 12.

**Table S1.** Serum biochemistry values (ITT).

## Data Availability

Research data are not shared.
