# Supplementary material for: Effects of Daily Kombu (Laminaria japonica) Intake on Body Composition, Blood Pressure, and Fecal Microbiota in Healthy Adult Japanese: A Randomized, Double‐Blind Study
Source: Food Sci Nutr. 2025 May 19;13(5):e70298. doi: 10.1002/fsn3.70298 (PMC12086367; doi:10.1002/fsn3.70298)
Supplement: Supplementary file 1 — Figure S1. Appearance of the test cookies. Figure S2. The average relative abundances of detected bacteria phylum and genera in stool samples obtained at baseline and week 12. Figure S3. Fecal SCFA concentrations of subjects at baseline and week 12. [file FSN3-13-e70298-s001.pptx]

## Slide 1
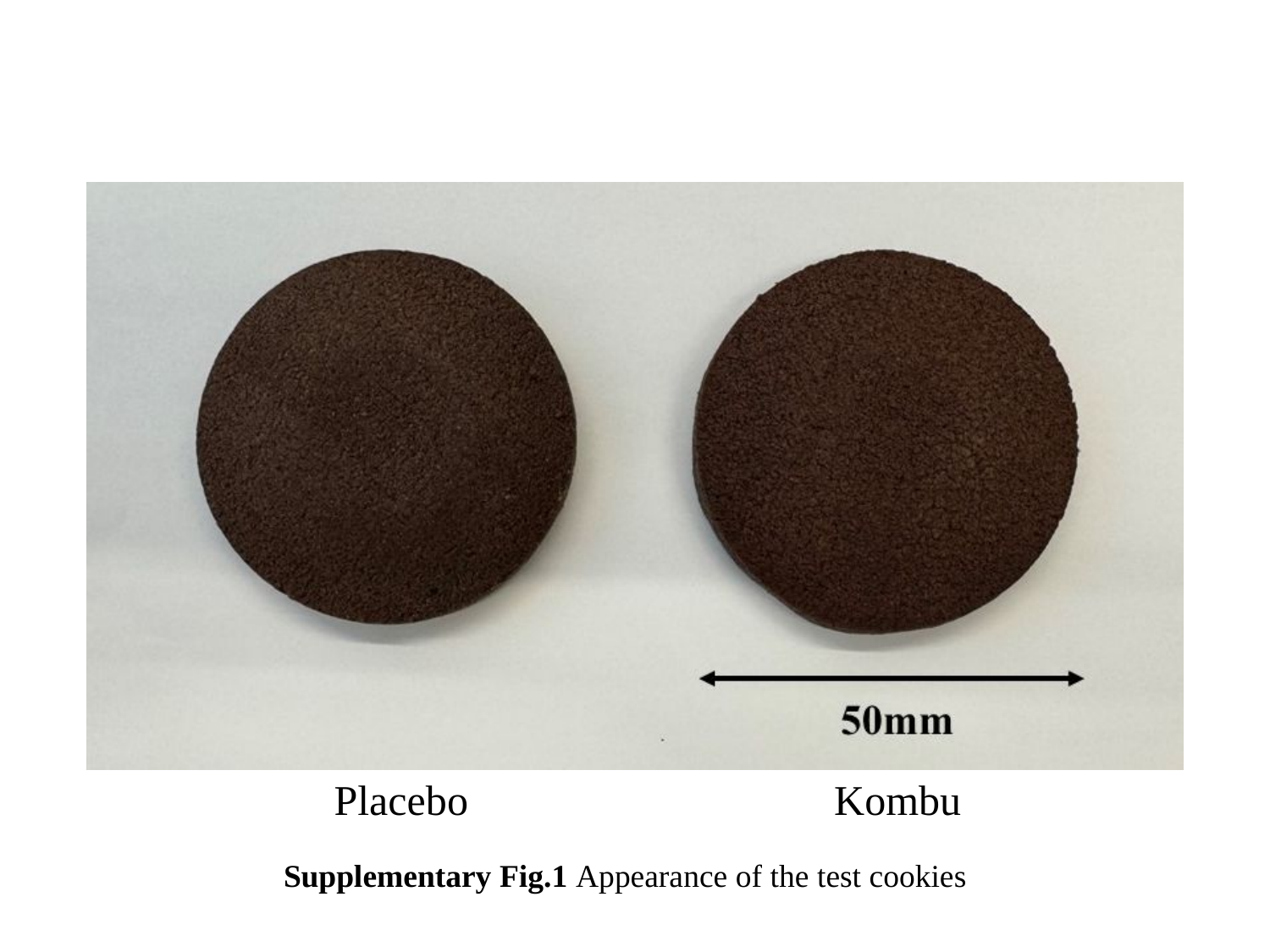

Kombu
Placebo
Supplementary Fig.1 Appearance of the test cookies

## Slide 2
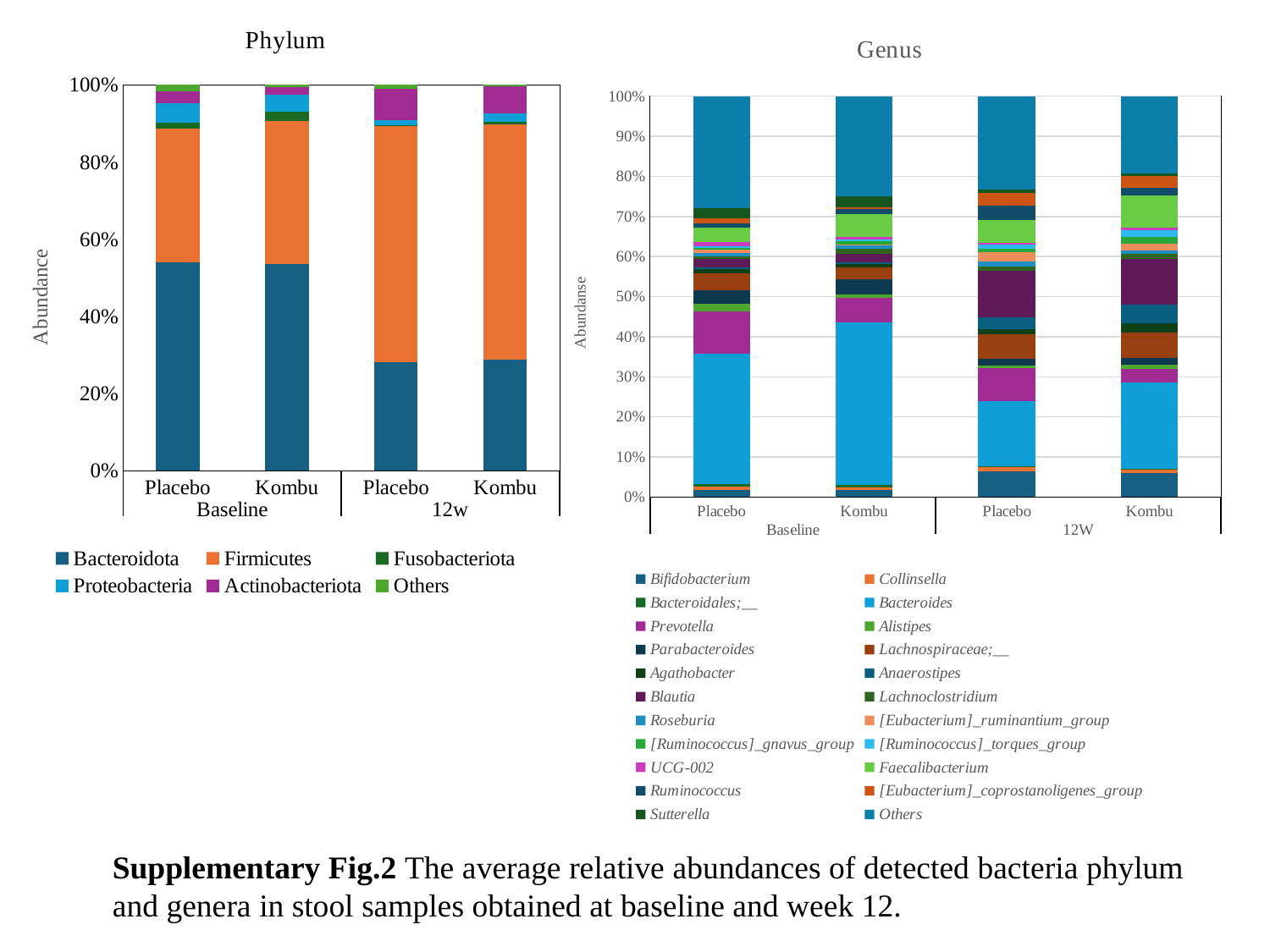

### Chart: Phylum
| Category | Bacteroidota | Firmicutes | Fusobacteriota | Proteobacteria | Actinobacteriota | Others |
|---|---|---|---|---|---|---|
| Placebo | 54.01095238095237 | 34.577619047619045 | 1.6304761904761902 | 5.007619047619047 | 3.1228571428571428 | 1.650476190476212 |
| Kombu | 53.490526315789474 | 37.19368421052633 | 2.232105263157895 | 4.4584210526315795 | 2.034736842105263 | 0.5905263157894609 |
| Placebo | 28.215954965037422 | 61.11770910400264 | 0.15348986512336818 | 1.4176735747242382 | 7.927255625644479 | 1.1679168654678591 |
| Kombu | 28.78310142682716 | 61.05559664940811 | 0.6511685566184472 | 2.0904153958274287 | 6.936667568518605 | 0.48305040280024514 |
### Chart: Genus
| Category | Bifidobacterium | Collinsella | Bacteroidales;__ | Bacteroides | Prevotella | Alistipes | Parabacteroides | Lachnospiraceae;__ | Agathobacter | Anaerostipes | Blautia | Lachnoclostridium | Roseburia | [Eubacterium]_ruminantium_group | [Ruminococcus]_gnavus_group | [Ruminococcus]_torques_group | UCG-002 | Faecalibacterium | Ruminococcus | [Eubacterium]_coprostanoligenes_group | Sutterella | Others |
|---|---|---|---|---|---|---|---|---|---|---|---|---|---|---|---|---|---|---|---|---|---|---|
| Placebo | 1.7353809575512773 | 0.8833451580766045 | 0.6294597983059196 | 33.31827276673266 | 11.005374541843148 | 1.8086123193020196 | 3.583560310733521 | 4.186110541175357 | 1.19602340050297 | 0.32798534201432633 | 2.143449374662839 | 0.8016901570537327 | 0.8038318284294206 | 0.7882297500558032 | 0.5365062816800042 | 0.44712796623921475 | 1.1379324344972093 | 3.5247874222892865 | 1.1552275673349275 | 1.2584068544156028 | 2.524026076392822 | 28.728685227104123 |
| Kombu | 1.8049888917431303 | 0.6613523222105646 | 0.7564139648555448 | 41.476322636116 | 6.284808944593768 | 1.016269593188805 | 3.838724414203296 | 3.1401833150672953 | 0.7744950612557656 | 0.485836045757141 | 2.0678302531642196 | 1.3671435327853607 | 0.9067961471678359 | 0.206448495607362 | 0.7422475947358939 | 0.5596989281887317 | 0.5904571625628852 | 5.939616467636297 | 1.2616443548255358 | 0.43937079549445474 | 2.7560023346776554 | 25.6793510788401 |
| Placebo | 6.399370805762374 | 1.2543099393301644 | 0.11805011601831202 | 16.465862834600596 | 8.29397289281283 | 0.6170689586667851 | 1.6064170310137609 | 6.228297729665227 | 1.210344199344818 | 3.0448747625736217 | 11.593101095177806 | 1.179758145704276 | 1.180841299942336 | 2.338152096229626 | 0.8741042181687353 | 0.9751139404055522 | 0.5249820243413679 | 5.714340884723116 | 3.7429840956041445 | 3.1174967760668926 | 0.7913616615168504 | 23.520556153847664 |
| Kombu | 6.053154378307868 | 0.9565838409999085 | 0.1830203074196507 | 21.638085041087265 | 3.354884735787067 | 1.095896286473229 | 1.561273842142251 | 6.4218704636992685 | 2.43965492801912 | 4.734966990467543 | 11.267606244458685 | 1.2812718793963986 | 1.0555596571099901 | 1.5425120023418384 | 1.7467732586126055 | 1.791832149430953 | 0.4920302098651573 | 8.164680853853538 | 1.9543708473868826 | 2.8616822417430163 | 0.6968831092530939 | 19.40228984139776 |Supplementary Fig.2 The average relative abundances of detected bacteria phylum and genera in stool samples obtained at baseline and week 12.

## Slide 3
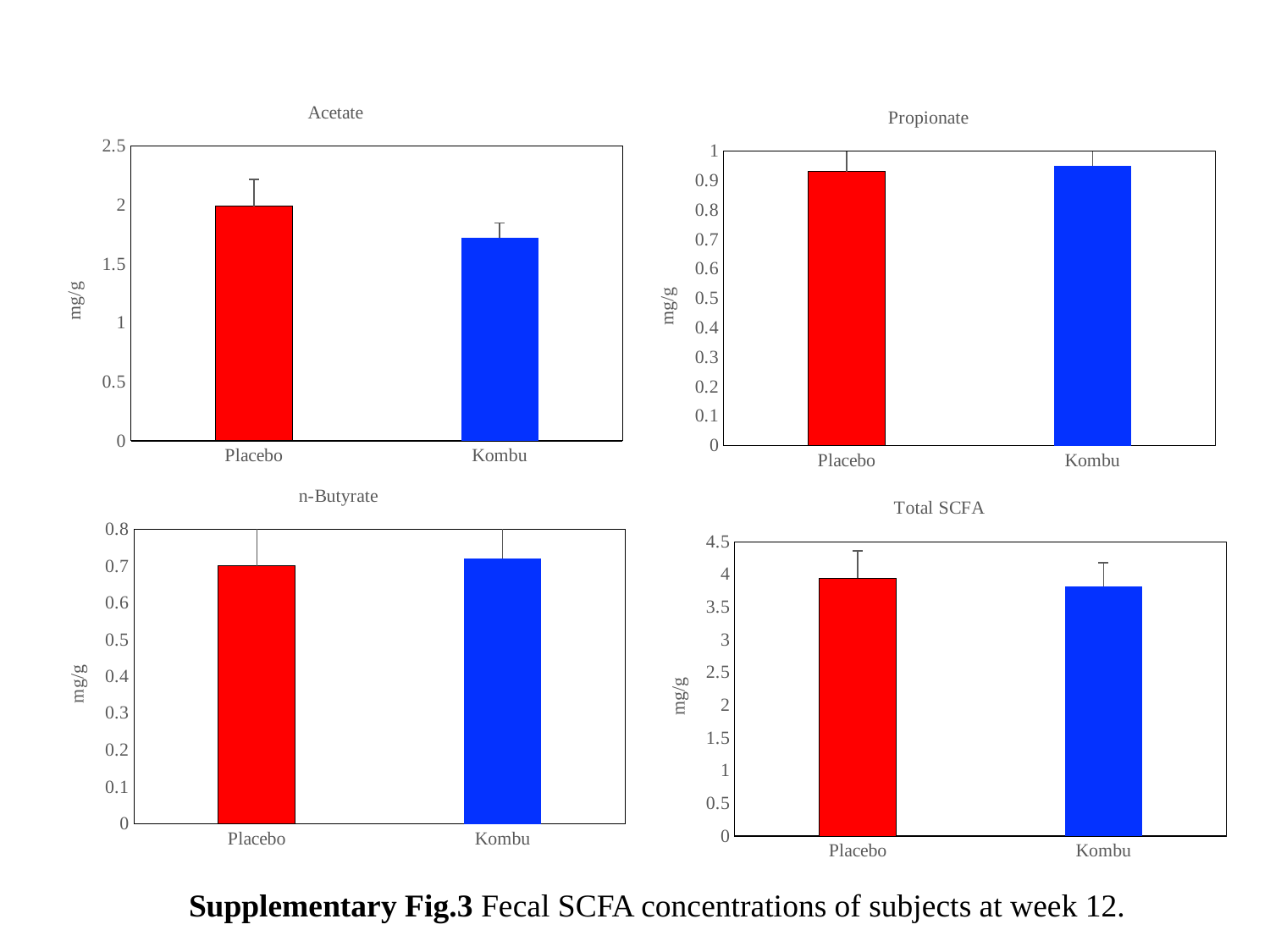

### Chart:
| Category | Acetate |
|---|---|
| Placebo | 1.99 |
| Kombu | 1.72 |
### Chart:
| Category | Propionate |
|---|---|
| Placebo | 0.93 |
| Kombu | 0.95 |
### Chart: n-Butyrate
| Category | n-butyrate |
|---|---|
| Placebo | 0.7 |
| Kombu | 0.72 |
### Chart:
| Category | Total SCFA |
|---|---|
| Placebo | 3.94 |
| Kombu | 3.81 |Supplementary Fig.3 Fecal SCFA concentrations of subjects at week 12.
