# Supplementary material for: Effects of Daily Kombu (Laminaria japonica) Intake on Body Composition, Blood Pressure, and Fecal Microbiota in Healthy Adult Japanese: A Randomized, Double‐Blind Study
Source: Food Sci Nutr. 2025 May 19;13(5):e70298. doi: 10.1002/fsn3.70298 (PMC12086367; doi:10.1002/fsn3.70298)
Supplement: Supplementary file 2 — Table S1. Serum biochemistry values (ITT). [file FSN3-13-e70298-s002.docx]

**Supplemental Table 1** Serum biochemistry values (ITT)

|  |  | baseline | | | 6W | | | 12W | | |  |
| --- | --- | --- | --- | --- | --- | --- | --- | --- | --- | --- | --- |
| WBC (10^9^/L) | Placebo | 5867 | ± | 255 | not determined | | | 5877 | ± | 291 |  |
|  | Kombu | 6938 | ± | 539 |  |  |  | 6007 | ± | 310 |  |
| RBC (10^12^/L) | Placebo | 494 | ± | 7 |  |  |  | 484 | ± | 7* |  |
|  | Kombu | 486 | ± | 8 |  |  |  | 476 | ± | 7* |  |
| Hb (g/L) | Placebo | 14.8 | ± | 0.3 |  |  |  | 14.7 | ± | 0.4 |  |
|  | Kombu | 14.6 | ± | 0.3 |  |  |  | 14.6 | ± | 0.3 |  |
| Ht (L) | Placebo | 45.6 | ± | 0.8 |  |  |  | 45.2 | ± | 0.9 |  |
|  | Kombu | 45.4 | ± | 0.7 |  |  |  | 45.3 | ± | 0.7 |  |
| Plt (10^9^/L) | Placebo | 28.5 | ± | 1.4 |  |  |  | 28.5 | ± | 1.4 |  |
|  | Kombu | 29.1 | ± | 1.3 |  |  |  | 29.4 | ± | 1.3 |  |
| TP (g/L) | Placebo | 7.34 | ± | 0.06 | 7.38 | ± | 0.05 | 7.28 | ± | 0.05 |  |
|  | Kombu | 7.30 | ± | 0.09 | 7.33 | ± | 0.09 | 7.23 | ± | 0.09 |  |
| Alb (g/L) | Placebo | 4.54 | ± | 0.04 | 4.54 | ± | 0.05 | 4.53 | ± | 0.05 |  |
|  | Kombu | 4.44 | ± | 0.04 | 4.39 | ± | 0.07 | 4.38 | ± | 0.07 |  |
| T-bil (μmol/L) | Placebo | 12.1 | ± | 1.0 | 11.0 | ± | 0.9 | 11.5 | ± | 0.9 |  |
|  | Kombu | 12.3 | ± | 0.8 | 12.5 | ± | 1.1 | 11.9 | ± | 0.8 |  |
| AST (U/L) | Placebo | 23.9 | ± | 1.1 | 25.3 | ± | 1.1 | 26.0 | ± | 2.4 |  |
|  | Kombu | 23.9 | ± | 1.5 | 24.0 | ± | 1.8 | 23.6 | ± | 1.9 |  |
| ALT (U/L) | Placebo | 25.4 | ± | 3.2 | 26.3 | ± | 3.0 | 28.6 | ± | 4.5 |  |
|  | Kombu | 26.1 | ± | 2.6 | 28.6 | ± | 5.3 | 26.5 | ± | 3.9 |  |
| ALP (U/L) | Placebo | 78.9 | ± | 4.0 | 76.4 | ± | 4.1 | 77.4 | ± | 5.1 |  |
|  | Kombu | 67.4 | ± | 2.2* | 64.8 | ± | 2.4 | 63.4 | ± | 2.2* |  |
| LD (U/L) | Placebo | 190.7 | ± | 5.4 | 189.5 | ± | 4.3 | 188.8 | ± | 5.0 |  |
|  | Kombu | 181.4 | ± | 6.0 | 177.2 | ± | 7.0 | 181.3 | ± | 7.8 |  |
| γ₋GT (U/L) | Placebo | 36.1 | ± | 5.0 | 37.0 | ± | 5.2 | 38.8 | ± | 6.7 |  |
|  | Kombu | 35.5 | ± | 4.9 | 35.3 | ± | 4.6 | 34.7 | ± | 4.2 |  |
| CK (U/L) | Placebo | 128.0 | ± | 14.9 | 163.4 | ± | 34.7 | 135.4 | ± | 15.3 |  |
|  | Kombu | 140.1 | ± | 27.8 | 137.5 | ± | 22.8 | 120.9 | ± | 17.2 |  |
| Total cholesterol (mmol/L) | Placebo | 5.81 | ± | 0.16 | 5.91 | ± | 0.15 | 5.63 | ± | 0.16 |  |
|  | Kombu | 5.83 | ± | 0.22 | 5.58 | ± | 0.37 | 5.62 | ± | 0.21 |  |
| HDL-cholesterol (mmol/L) | Placebo | 1.56 | ± | 0.08 | 1.51 | ± | 0.07 | 1.48 | ± | 0.07^*^ |  |
|  | Kombu | 1.61 | ± | 0.08 | 1.51 | ± | 0.11 | 1.50 | ± | 0.08^*^ |  |
| LDL-cholesterol (mmol/L) | Placebo | 3.69 | ± | 0.11 | 3.75 | ± | 0.12 | 3.44 | ± | 0.12^*^ |  |
|  | Kombu | 3.64 | ± | 0.16 | 3.51 | ± | 0.25 | 3.37 | ± | 0.15^*^ |  |
| Triglyceride (mmol/L) | Placebo | 1.36 | ± | 0.17 | 1.46 | ± | 0.18 | 1.42 | ± | 0.18 |  |
|  | Kombu | 1.36 | ± | 0.16 | 1.25 | ± | 0.18 | 1.67 | ± | 0.33 |  |
| Glucose (mmol/L) | Placebo | 5.22 | ± | 0.08 | 5.36 | ± | 0.09 | 5.44 | ± | 0.10^*^ |  |
|  | Kombu | 5.16 | ± | 0.09 | 5.05 | ± | 0.29 | 5.31 | ± | 0.10 |  |
| HbA1c (%) | Placebo | 5.40 | ± | 0.08 | 5.43 | ± | 0.06 | 5.42 | ± | 0.05 |  |
|  | Kombu | 5.58 | ± | 0.04 | 5.50 | ± | 0.07 | 5.47 | ± | 0.08 |  |
| Uric acid (μmol/L) | Placebo | 322.4 | ± | 16.4 | 355.1 | ± | 18.4^*^ | 348.0 | ± | 20.9 |  |
|  | Kombu | 348.9 | ± | 15.0 | 354.6 | ± | 23.0 | 353.5 | ± | 16.5 |  |
| BUN (mmol/L) | Placebo | 4.44 | ± | 0.35 | 4.55 | ± | 0.34 | 4.22 | ± | 0.32 |  |
|  | Kombu | 4.57 | ± | 0.21 | 4.43 | ± | 0.32 | 4.72 | ± | 0.26 |  |
| Creatinine (μmol/L) | Placebo | 71.0 | ± | 3.4 | 73.5 | ± | 3.3 | 66.7 | ± | 3.4^*^ |  |
|  | Kombu | 75.9 | ± | 3.8 | 74.0 | ± | 5.2 | 70.4 | ± | 3.4^*^ |  |

No significant differences between the placebo and kombu groups were observed at baseline, 6W, and 12W (Bonferroni’s multiple comparison). *: p<0.05 (vs baseline, Dunnett’s multiple comparison) Mean ± standard error (SE)

Abbreviations: WBC; white blood cell, RBC: red blood cell, Hb; hemoglobin, Ht; hematocrit, Plt; platelet, TP; total protein, Alb; albumin, T-bil; total bilirubin, AST; aspartate aminotransferase, ALT; alanine aminotransferase, ALP; alkaline phosphatase, LD; lactate dehydrogenase, γ-GT; γ-glutamyltransferase, CK; creatine kinase, HbA1c; hemoglobin A1c (NGSP;National Glycohemoglobin Standardization Program)BUN; blood urea nitrogen)
